# Is the computed tomography exam important for planning mini-implant installation?

**DOI:** 10.4317/jced.60288

**Published:** 2023-04-01

**Authors:** Eduardo-Silveira Rodrigues, Carolina-Morsani Mordente, Lizandra-Gonzaga Rodrigues, Izabella-Lucas de Abreu Lima, Diogo-de Azevedo Miranda, Elton-Gonçalves Zenóbio, Flávio-Ricardo Manzi

**Affiliations:** 1 Department of Dentistry, Pontifical Catholic University of Minas Gerais, Belo Horizonte, Minas Gerais, Brazil

## Abstract

**Background:**

Mini-implants are devices used to provide absolute and temporary anchorage for tooth movement. Objectives: The present study was carried out to compare the use of periapical radiographs and computed tomography (CT) for planning mini-implants performed by orthodontists.

**Material and Methods:**

Five radiographs and five CT scans of premolars and molars regions. These were analyzed by ten Orthodontists. Initially (T1), the evaluators indicated the preferred location for the insertion of a mini-implant, as well as the diameter and length of the device, using only a periapical radiograph. After 30 days (T2), the same evaluation was performed. Sixty days later (T3), the orthodontists reassessed the radiographs in association with the CT scans. Finally, after 90 days (T4), the evaluation was performed with the same exams. The comparison of the chosen diameter and length of the mini-implants was performed using the Student’s t-test. The evaluation of the chosen insertion sites was analyzed by the Wilcoxon test. For both tests, the level of significance was 5%. The kappa concordance test was also performed for the intra- and inter-examiner evaluations.

**Results:**

The results of the study showed substantial or perfect intra-examiner and reasonable to perfect inter-examiner agreement. Considering the length and diameter of the mini-implants, no statistical difference was found between the groups. Regarding the insertion site, more than 20% of the treatment plans were changed when the CT scan was associated.

**Conclusions:**

The results showed that the association of a CT scan with radiography frequently leads the professional to change the insertion point for the installation of mini-implants.

** Key words:**Orthodontic anchorage procedures. Mini Dental Implants. Bone Screws. Cone-beam computed tomography. Periapical radiography.

## Introduction

Mini-implants are devices used to provide absolute and temporary anchorage for tooth movement. Anchorage is the resistance to unwanted tooth movement, and is mandatory for orthodontic treatment of malocclusions. Absolute anchorage is present when the anchorage unit does not move as a result of the reaction of the force applied to move the tooth, enabling a maximization of the desired tooth movement (1,2). In addition to absolute, mini-implants also provide temporary anchorage, since they can be easily removed when the desired orthodontic movement is completed (2). Because of these characteristics, orthodontic treatments using mini-implants can overcome limitations of conventional treatments and their use has steadily increased in orthodontic practice (3).

Other devices can be used in order to provide absolute anchoring, such as osseointegrated implants, temporary osseointegrated implants in the palatal suture and titanium miniplates. Despite these possibilities, mini-implants are the most widely used resource for this purpose due to their versatility, low invasiveness, low cost, and easy insertion and removal (4,5).

Despite presenting several advantages, mini-implants may offer some risks that should not be overlooked. Lesions in dental roots and low stability caused by contact with the root are examples of common complications during the insertion of these devices that can be avoided (5). The major cause of these complications during the insertion of the mini-implants is the lack of precise knowledge of the anatomy of the insertion area (3). Thus, it is essential to perform a good pre-surgical planning to avoid complications and increase the success rate of therapy.

To analyse the mini-implant insertion area, the surgeon often uses conventional radiographs. The two-dimensional (2D) evaluation of a three-dimensional (3D) area increases the chances of diagnostic errors and, consequently, accidents (3). Recent advances in 3D imaging techniques (computed tomography (CT)) have enabled dentists to overcome these limitations and considerably reduce the rate of complications (3,5,6). One study, evaluating the use of surgical guides for the insertion of mini-implants, showed that 52.3% of the guides made based on conventional radiographs had to be modified after the study of the same case using a computed tomography (6).

Despite the evidence found in the literature regarding the limitations of traditional radiographs, many professionals choose to install mini-implants without using CT scans for planning. Hence, this study aims to evaluate the chosen insertion site when the professional has only access to the radiography of the insertion area and when he has access to the radiography and CT of the same area.

## Material and Methods

The present study was submitted and approved by the Research Ethics Committee, in [localization omitted for blinded review]. After its approval (logged under protocol number: [number omitted for blinded review]) the procedures described below were carried out.

-Sample

Five periapical radiographs and five CT scans of maxillary premolars and molars were used. Thus, a total of five pairs of corresponding images from the same area and patient were used for the evaluation. Each pair was evaluated by ten orthodontists, totaling 50 evaluation points.

-Acquisition of images

Periapical radiographs were performed with the Kodak 2200 Intraoral X-Ray System® radiograph (Carestream Health, Inc., Rochester, New York, USA) and parallelism technique. For the PSP system, the Scan-X Duo (Air Techniques, Inc., Melville, New York, USA) digital sensor was used. All radiographs were taken by specialists in dental radiology. Exposure factors (exposure time, milliamperage and kilovoltage) were determined according to the manufacturer’s recommendations. The cone-beam computed tomography (CBCT) scans were performed using the CS 8100-3D (Carestream Dental, Rochester, New York, USA), 140kHz, 60kV, 2mA, 75um Voxel and 5.0cm x 5.0 cm FOV. The digital images were stored in the Kodak Dental Imaging Software (KDIS) system (Carestream Health Inc., Rochester, New York, USA) in the original format (DICOM) with the individuals’ codes.

-Image analysis

Initially, all radiographic and tomographic images were coded by the main researcher, who did not perform any of the analyses. All images were analysed by ten orthodontists with experience in mini-implants, at four different times (T1, T2, T3, T4). For the analysis, orthodontists were previously trained in the tomography software. The evaluations were made on a computer containing a GeForce 9500 GT graphics card (Nvidia Corporation, Santa Clara, California, USA) and an LG Flatron E2241 LED monitor (LG Electronics, Seul, South Korea), with a resolution of 1920x1080 pixels. The levels of brightness and contrast were fixed in their pre-established conFiguration. To offer the same conditions to the observers, the evaluations were made in the same place, with the same computer and monitor.

**Figure 1:**
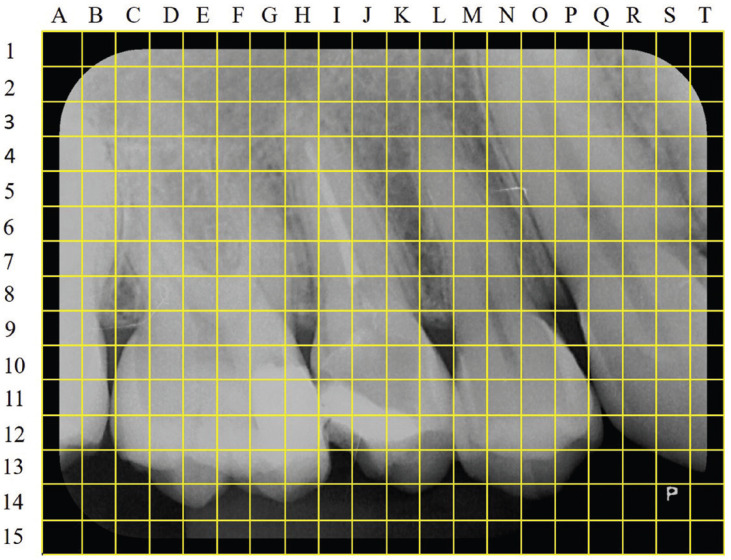
Illustration of response matrix to choose the best insertion point for a miniscrew.

In the first stage (T1), orthodontists analysed only digital periapical radiographs. They were instructed to choose the best insertion point for a mini-implant with an angle of 0 degrees in order to perform the movement to retract the patient’s anterior battery. They also indicated the diameter and length of the mini-implant they would choose for this purpose. The diameter options were 2.0 mm, 1.5 mm and 1.3 mm, together with a length of 8 mm, 10 mm or 12 mm. As a template, an answer sheet was made up of a schematic Figure of the periapical radiography, where orthodontists marked the ideal insertion site (Fig. 1). The chosen diameter and length were noted on the same sheet. Thirty days after the first evaluation (T2), the same orthodontists analysed the periapical radiographic images again and answered the same questions in a new answer sheet. This measure was made to minimize vicious analyses and evaluate intra-examiner responses.

**Figure 2:**
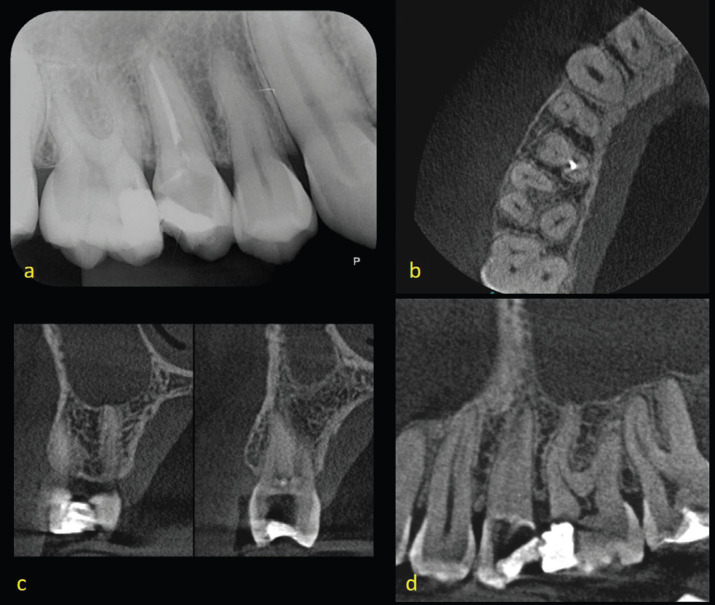
Analized images: radiograph and slices of CT scans of the same region. (a) radiograph, (b) axial, (c) parasagittal, (d) sagital.

After sixty days (T3), orthodontists analysed the radiographic images, associated with CT scans, of the same region (Fig. 2). In another answer sheet, they answered the same questions asked previously. Finally, after 90 days (T4), another analysis of the radiographs associated with CT scans, was performed. This evaluation interval is important so as to minimize the possibility of bias.

The answer sheet contains a schematic picture of each periapical radiograph. In it, lines and columns with 2 mm spacing between the intersection points were drawn, forming subareas with dimensions of 4 mm2 (2 mm x 2 mm). These sub-areas were named according to the axes that make up their height and width, with the height axis in sequence of Arabic numerals and the width axis in alphabetical sequence (Fig. 1). Thus, the evaluators marked which subarea of the periapical radiography considered the best position for the installation of the mini-implant, at the two moments of evaluation (only with the digital periapical radiography and with the association of the digital periapical radiography and the CT scan).

After performing radiographic and tomographic analyses, the kappa intra- and inter-examiner concordance test was performed. The comparison of the chosen diameters and lengths was made using the Student’s t-test. The evaluation of the chosen insertion points was analysed by the Wilcoxon test.

## Results

According to the unweighted kappa test (Table 1), the inter-observer assessment values ranged from reasonable to perfect agreement (0.21; 0.85), and proved to be reasonable for Patient 2, moderate for Patient 4, substantial for patients 1 and 5, and perfect for Patient 3. In the intra-observer analysis, the agreement of the results was substantial or perfect (0.8-0.9) for most patients, with the exception of Patient 2, who showed a moderate agreement (0.60). The kappa test was also used to analyze the inter- and intra-examiner variation of the mini-implant lengths and diameter, with k = 0.88 / 0.94 and k = 0.92 / 0.96 respectively, which demonstrates an excellent level of agreement.

**Table 1:**
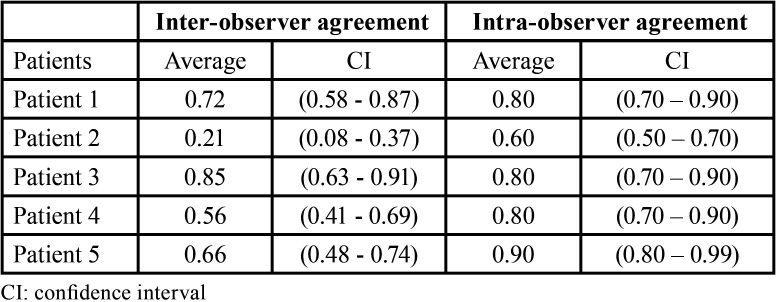
Unweighted kappa test (95% confidence interval (CI)) for intra- and inter-observer agreement for the five proposed clinical cases.

As for the length and diameter of choice, no statistical difference was found between the two groups (Table 2).

**Table 2:**
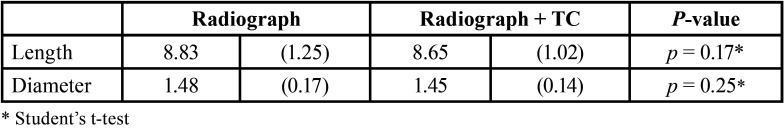
Average (and standard deviation) of the mini-implant lengths and diameters, in millimeters, chosen by professionals using only periapical radiographs and an association of periapical radiographs with a CBCT scan.

However, in Table 3, it can be seen that more than 20% of the treatment plans regarding the location of mini-implants were modified when the association of periapical radiography with CT scans was used. This value represents a statistically significant difference in the choice of the positioning of mini-implants when comparing the use of only periapical radiographs with their association with CT images (*p* <0.05%).

**Table 3:**
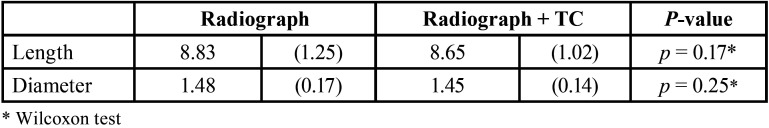
Absolute numbers and percentage of coincident mini-implant locations using only periapical radiography and the association of radiography and CBCT scans.

## Discussion

Achieving excellent dentistry requires professionals to work with the least margin of error possible. Each day, patients are more aware of the different diagnostic and treatment options available, as well as more demanding with the results obtained. Seeking to achieve health and patient satisfaction, professionals must use all available resources to minimize errors and complications. Proper planning is a key to the success of therapy and, to achieve this, it is imperative to obtain the correct diagnosis of the case.

The use of mini-implants for orthodontic anchorage is a consolidated practice and is well supported by the literature (2,7-9). Despite the high success rate obtained with the use of these devices, complications during installation and orthodontic movement are not uncommon (5). A good anatomical knowledge of the surgical area and an adequate planning of the case are important to avoid complications that occur frequently, such as contact of the device with dental roots during its installation (3,5,6,10-12).

In clinical practice, it is common to plan the installation of mini-implants with the exclusive use of radiographs. This examination, however, when providing a 2D image of a 3D region, generates overlaps and distortions of structures, which can make diagnosis and proper surgical planning difficult. By contrast, CBCT scans provide a 3D image, with minimal values of distortion and overlap (13). The present study was conducted with the objective of evaluating the importance of CT images in planning the installation of mini-implants. For this purpose, the diameter, length and insertion point chosen for this procedure were analysed when the dentist has access to periapical radiography and when he has access to CT scans associated with radiography.

The results of this study showed no statistically significant differences between the two groups in relation to the diameter and length chosen for the mini-implant. Although there are several options for device sizes on the market, there is a certain convergence in the literature on the lengths and diameters to be chosen. Screws with reduced diameter and length are more susceptible to fracture and stability failure and are therefore seldom used. Exaggerated measures are also rarely recommended, since they increase the risk of complications without generating benefits that justify their use (1). With few and restricted options recommended in the literature, the absence of a significant difference between groups is justified.

Regarding the chosen insertion point, more than 20% of the sites were altered when the association between radiography and CT scans was used, as compared to the group using only radiography. This result shows that the availability of CT scans often leads professionals to change their planning. This information converges with a previous work, in which it was demonstrated that 52.3% of the guides for the insertion of mini-implants made on the basis of conventional radiographs had to be modified after the reassessment of the same case using a CT scan (6).

Regarding the intra-examiner agreement test, a substantial or perfect result (0.8-0.9) was obtained for most patients, with the exception of patient 2, who showed a moderate agreement (0.60). This result, which is generally satisfactory, shows good consistency in the assessments made by the same examiner, which reinforces the validity of the obtained data. The inter-examiner concordance test showed greater variations in relation to the intra-examiner. The values obtained ranged from reasonable to perfect agreement (0.21; 0.85), and proved to be reasonable for patient 2, moderate for patient 4, substantial for patients 1 and 5, and perfect for patient 3. This test shows that in certain situations there was a greater divergence in the treatment plan chosen by the evaluators. This can be explained by the different treatment options available for the same case, as well as by the varied philosophies of work that different professionals may have. In some clinical situations, especially in the most complex cases, variations in the solutions to perform the desired orthodontic movement are common.

Not long ago it was common to plan the installation of dental implants using panoramic and periapical radiographs. However, scientific evidence has shown the various deficiencies of these exams for this purpose (13). Thus, the current consensus is that radiographic examinations should not be used alone for dental implant planning. Currently, CT scans are considered essential and are routinely used for the proper planning of osseointegrable implants (13). The present study shows a similar situation for the installation of mini-implants. According to the results obtained, 20% of the insertion points chosen by the professionals through the use of radiographs were changed when the CT scan was added to the case planning.

This expressive value demonstrates the high risk of error to which dental professionals are exposed when performing this procedure without using a CT scan as a diagnostic tool. By providing 3D images, without overlapping and with a very low distortion, the tomographic examination reduces the risk of failure in planning the installation of mini-implants. For this reason, CT scans should be indicated as routine tools for this purpose. This conduct will benefit both patients and dental professionals, as it reduces the risks of accidents and complications.

## Conclusions

Taking into account the design of the present study, it can be concluded that the association of CT scans with periapical radiography often leads the dental professional to change the insertion point for the installation of mini-implants, when compared to the planning performed only with 2D exams. Thus, the routine use of CT scans is recommended when planning the installation of these devices.
